# Experimental screening studies on rabies virus transmission and oral rabies vaccination of the Greater Kudu (*Tragelaphus strepsiceros*)

**DOI:** 10.1038/s41598-018-34985-5

**Published:** 2018-11-09

**Authors:** Rainer Hassel, Ad Vos, Peter Clausen, Susan Moore, Jolandie van der Westhuizen, Siegfried Khaiseb, Juliet Kabajani, Florian Pfaff, Dirk Höper, Boris Hundt, Mark Jago, Floris Bruwer, Pauline Lindeque, Stefan Finke, Conrad M. Freuling, Thomas Müller

**Affiliations:** 1 Present Address: School of Veterinary Medicine, University of Namibia, Private Bag 13301, Windhoek, Namibia; 2 Present Address: IDT Biologika GmbH, Am Pharmapark, 06861 Dessau-Rosslau, Germany; 3 Okosongoro Safari Ranch, P.O. Box 324, Omaruru, Namibia; 4 Kansas State University, Veterinary Diagnostic Laboratory, Rabies Laboratory, Manhattan, KS 66502 USA; 5 Central Veterinary Laboratory, Private Bag 13187, Windhoek, Namibia; 6 Institute of Diagnostic Virology, Friedrich-Loeffler-Institute, Südufer 10, 17493 Greifswald - Insel Riems, Germany; 7 ProVision at Agra Ltd., Private Bag 12011, Windhoek, Namibia; 8 Institute of Molecular Virology and Cell Biology, Friedrich-Loeffler-Institute, Südufer 10, 17493 Greifswald - Insel Riems, Germany

## Abstract

Rabies in the Greater Kudu (*Tragelaphus strepsiceros)* in Namibia is unique and found in such magnitude as has not been reported elsewhere in southern Africa. Reasons as to why Kudus appear to be exceptionally susceptible to rabies still remain speculative at best. Because the current severe rabies endemic in Kudus continues to have an enormous negative impact on the Namibian agricultural sector, we set out to question existing dogmas regarding the epidemiology of the disease in a unique experimental setting. In addition, we explored effective measures to protect these antelopes. Although we were able to confirm high susceptibly of kudus for rabies and sporadic horizontal rabies virus transmission to contact animals, we contend that these observations cannot plausibly explain the rapid spread of the disease in Kudus over large territories. Since parenteral vaccination of free-roaming Kudus is virtually impossible, oral rabies vaccination using modified life virus vaccines with a high safety profile would be the ultimate solution to the problem. In a proof-of-concept study using a 3rd generation oral rabies virus vaccine construct (SPBN GASGAS) we found evidence that Kudus can be vaccinated by the oral route and protected against a subsequent rabies infection. In a second phase, more targeted studies need to be initiated by focusing on optimizing oral vaccine uptake and delivery.

## Introduction

Rabies in the Greater Kudu (*Tragelaphus strepsiceros*) in Namibia is unique and occurs in such magnitude as has not been reported outside Namibia nor does it affect other game species in southern Africa to the same extent^1^. Kudu rabies was not known before the mid - 1970s. Until this point in time only sporadic but endemic rabies was reported throughout most of Namibia mainly in dogs, livestock and wildlife, though geographically separated^2–5^. In 1975, the first cases of rabid Kudus were detected in central Namibia near Windhoek. From here, the disease spread northwards to all major habitats of Kudus in the country, including the Etosha National Park^6,7^, though this assumption may be biased by enhanced awareness and surveillance. During this first epidemic event, however, that lasted from 1977 to 1986 an estimated 50,000 Kudus, approximately 20% of the total population, had succumbed to rabies. In the following years only isolated sporadic outbreaks in Kudus were reported. This changed in 2002, when another sharp increase in Kudu rabies cases was recorded^8^. Today, rabies in Kudus is endemic affecting a large portion of both the Kudu population and habitat in Namibia^9^.

Reasons as to why Kudus appear to be exceptionally affected by the disease still remain speculative at best. Initially, it was assumed that spill-over infections from rabies-infected jackals were the cause of the rabies cases in Kudus^1,10^. Later the possibility of independent horizontal transmission among Kudus was suggested as a result of social - and feeding behavior of this animal species^11–13^.

Kudu herds typically consist of 1 to 3 females with their offspring. However, herds may temporarily merge forming groups of up to 30 animals. Males form transient bachelor groups that can include mature bulls after the main mating season^14^. One of the most commonly observed symptoms of a rabies infection in Kudus is hyper-salivation^12^. Because the animals often browse simultaneously from the same shrub or tree and have close contact with each other through activities such as mutual social licking, they could potentially come into contact with saliva containing rabies virus from an infected animal within the group. The assumption of horizontal transmission was further supported by early transmission experiments demonstrating that Kudus could become infected when infectious saliva was administered by the oral route^12^. Also, based on several unique mutations complete genome sequencing revealed rabies virus (RABV) isolates from Kudus to be distinct from those of dogs, but also from other terrestrial carnivores, like jackals, in Namibia further supporting the assumption that rabies is being maintained independently in the Namibian Kudu population^15^. Although anecdotal evidence apparently supports the hypothesis of horizontal rabies transmission among Kudus, it still remains a mystery whether this mode of transmission can really explain the recent outbreaks and apparent rapid spread of the disease over large territories.

Kudu rabies is a scourge for game farmers as the Namibian economy relies extensively on Kudu through the means of trophy hunting, game meat hunting and eco-tourism^1,8,16^. This browser is the second-largest antelope; bulls can weigh up to 315 kg^14^. Besides occurring naturally over much of eastern and southern Africa, Kudus are utilized in a number of ways such as (i) capture and sale of live animals for breeding purposes, (ii) trophy hunting and (iii) hunting for venison. To this end, Kudus are kept in fully fenced game farms, fenced commercial livestock/game farms, where livestock (usually cattle or small ruminants) and Kudus roam together and in enclosures (>2000 ha) on many game farms across southern Africa, in particular Namibia. Kudus have the third largest asset value of all farmed animals in Namibia. In 2004, the estimated value of Kudus was 31.13 million US dollars, which was greater than the total value of sheep, goats and donkeys combined^1^. Pre-exposure vaccination of Kudus against rabies seems a justified approach considering their economic value and observed high rabies-associated mortality. However, herding these animals by helicopter or vehicle into a holding facility for vaccination by intramuscular injection is extremely costly, labor intensive and a dangerous operation (stress induced injuries and/or mortality). Vaccination of free-living Kudus through darting from a vehicle has been proven over many years to be virtually impossible due to animals fleeing or going into hiding when vehicles and humans are observed. Hence, presently the only available option is vaccination by darting from a helicopter but this is very difficult, expensive and time consuming. In addition, Kudus have the habit of taking shelter under trees when they perceive an unknown noise such as a helicopter. The observation of artificial transmissibility of RABV by the oral route under experimental conditions^12^ led to the suggestion that if animals could become infected by the oral route, this route may also be feasible for vaccination purposes as was shown initially for the North American subspecies of the red fox (*Vulpes vulpes fulva*)^17^. Oral rabies vaccination of foxes and other mesocarnivores acting as reservoir species has been developed into the method of choice for rabies control in these species^18^.

Hence, the study presented here focused on (i) investigating the possibility of natural horizontal transmission of rabies among Kudus and (ii) exploring the proof-of-concept of oral vaccination of a herbivorous species against rabies. As regards the latter we were interested to see whether a genetically modified oral rabies vaccine with a high safety profile licensed for wild carnivores is able to induce a protective immune response in Kudus after direct oral application (DOA). The results were compared to animals parenterally immunized with a commercial inactivated rabies vaccine for livestock that served as a positive control group.

## Material and Methods

### Animals and housing conditions

**Figure 1:**
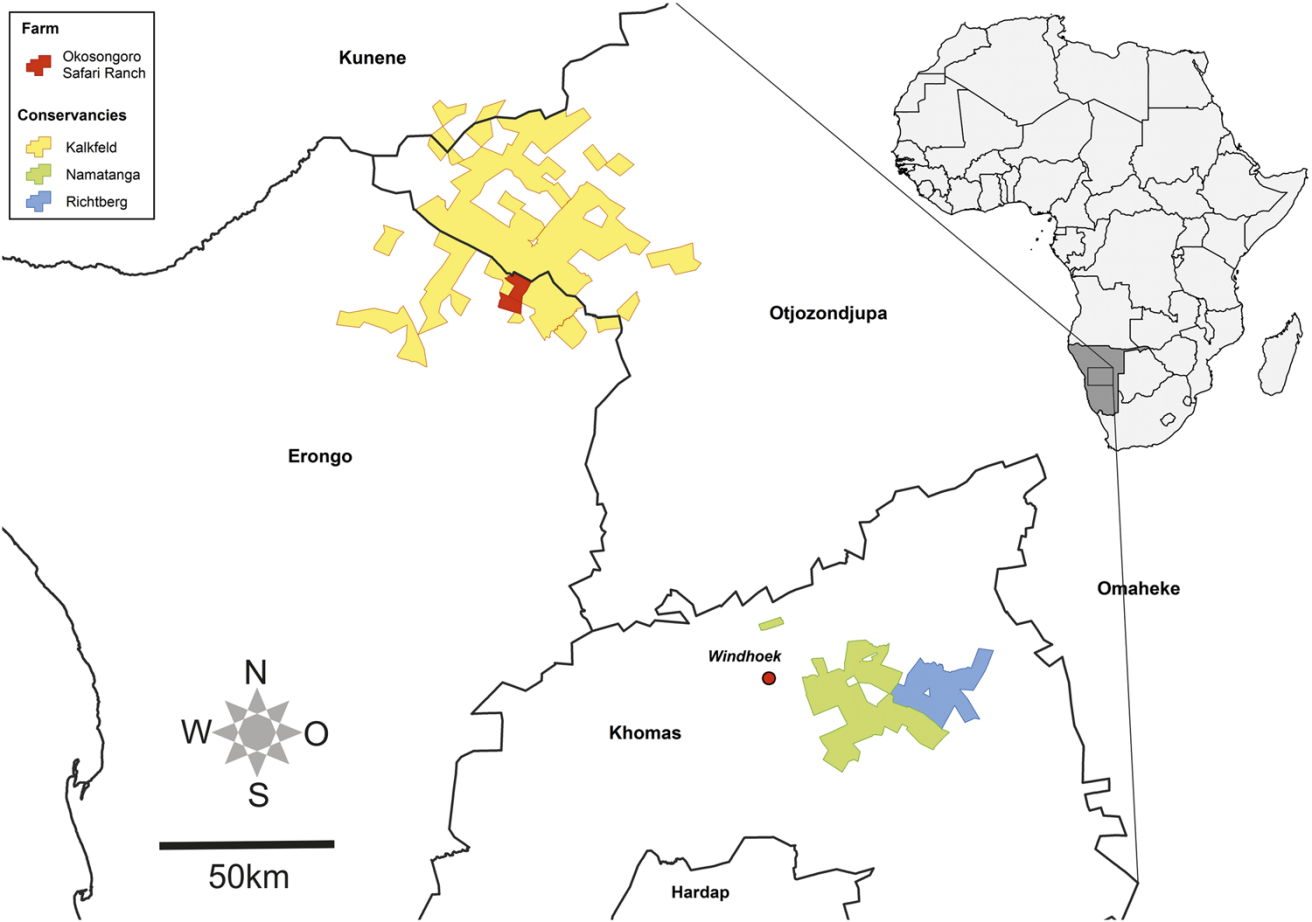
To the right: Map depicting the location of Namibia in Africa (right, dark grey). To the left: Enlargement of the middle regions of Namibia showing the locations of the three conservancies the Kudus were captured and of the experimental holding facility; 265 km northwest of Windhoek. Names of the Namibian districts are indicated.

Adult free-living Kudus (n = 46) were caught by mass capture or by individual darting from a helicopter on game farms in three different Conservancies and transported to the experimental holding facility on the Okosongoro Safari Ranch located about 265 km northwest of Windhoek (Fig. 1). Blood samples were taken on the day of capture to assess the immunological status of the animals.

**Figure 2:**
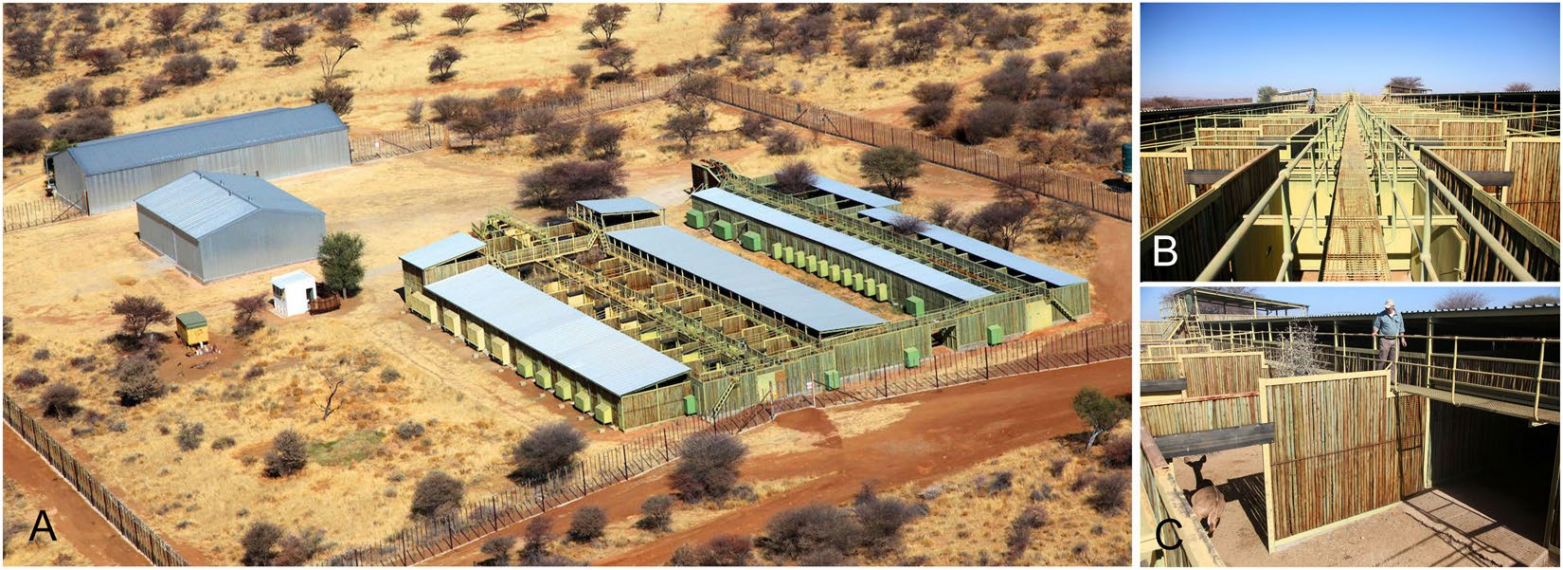
Aerial photography of the experimental holding facility at the Okosongoro Safari Ranch showing the individual pens as well as the supply buildings and staff quarters (**A**). To prevent contact with free-roaming wildlife from adjacent areas, the entire experimental holding facility and surrounding area was secured by a single 3.2 m high wire mesh game proofed fence. Overhead catwalks enabled staff to better observe and manipulate animals (**B**). Individual pen (“boma”) (7 m × 14 m) consisting of a covered - and an open area (**C**). The sliding door could be used to connect the pen with an adjacent pen for separating single animals. The height of the indoor part of the pen was 3 m and the outdoor section was surrounded by a 3 m high wall.

Separate groups of 4 to 6 animals of the same gender were housed in two attached pens (“boma”) (7 m × 14 m). Each boma consisted of a covered - and an open area which could be separated from each other by a sliding door. Both areas could be entered separately. Also, a sliding door could be used to connect the pen with an adjacent pen for separating single animals. The height of the indoor part of the pen was 3 m and the outdoor section was surrounded by a 3 m high wall. Large doors opening in both directions connect the pens with the central passage. Overhead catwalks enabled the staff to observe animals and facilitated the separation and manipulation of animals, e.g. immobilization for vaccination, infection and blood sampling. For the purposes of this study, to prevent contact with free-roaming wildlife from adjacent areas, the entire experimental holding facility and surrounding area was secured by a single 3.2 m high wire mesh game proofed fence (Fig. 2). Furthermore, no other animals were held in the experimental facility during the entire study period. The site was guarded 24 h per day.

### Animal feeding and prophylactic treatment

Upon arrival, the Kudus were treated with doramectin (Dectomax −1% solution, Pfizer, Sandton, South Africa) against internal and external parasites and received multivitamin preparations (Kyrovite B Co Super, Kyron, Johannesburg, South Africa) and metabolic stimulant injections (Kyrophos Metabolic-V, Kyron). During the period of confinement animals received a diet of lucerne straw (*Medicago sativa*) and game cubes (Boskos WESenterprises, Thabazimbi, South Africa) supplemented with pods of the camel thorn tree (*Acacia erioloba*) and umbrella thorn tree (*Acacia tortilis*). When the supply of Boskos was exhausted it was replaced by Standard Game Cubes alternated with Game Cubes plus Ivermectin (Feedmaster, Windhoek, Namibia). The provision of acacia pods was particularly reduced during the hot dry season when these fruit became unobtainable. Pens were cleaned daily by removing both droppings and uneaten feed spilled on the ground to minimize fly infestation as well as internal parasite burdens. Drinking water was offered *ad libitum* and supplemented at intervals with probiotics (Protexin, Kyron, Benrose, South Africa), minerals and vitamins (Game Min, Oudshoorn, South Africa).

### Transmission studies

**Figure 3:**
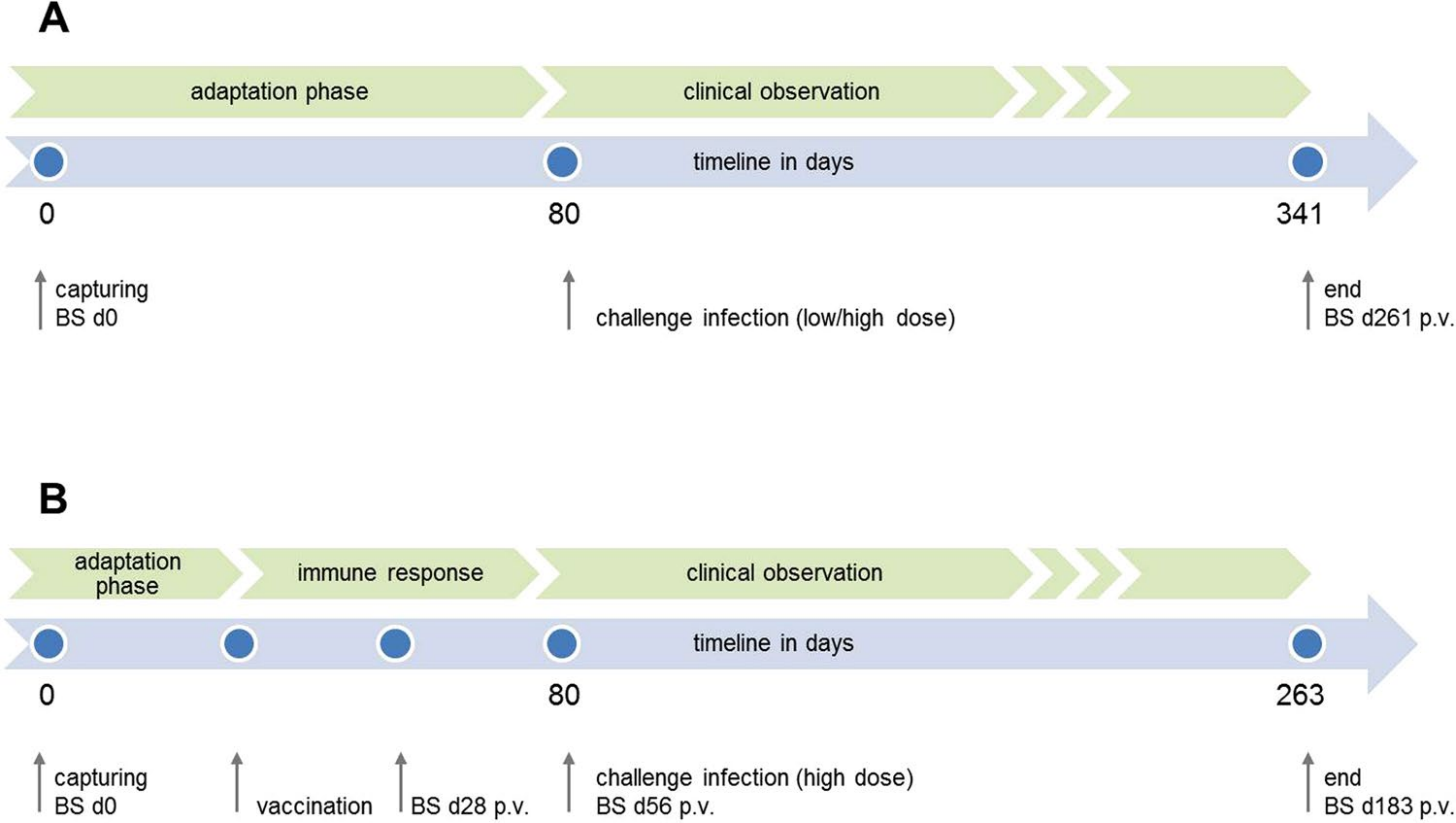
Figure displaying the experimental design for both the transmission (**A**) and the vaccination studies (**B**) as a timeline of events and planned target. The blue dots represent individual time points of interventions in terms of capturing, vaccination, challenge and blood sampling (BS).

For transmission studies, in total 21 Kudus (Table 1) were held in one group of six animals (group A) and 3 groups of five animals each (groups B-D, Table 2). Prior to infection, animals were adapted to captivity for an 80 days period. One animal in each group was infected with a high (10^5.3^TCID_50_, N = 4) dose, while another animal was inoculated with a low (10^3.3^ TCID_50_, N = 4) dose of a Kudu rabies virus isolate (see below). The remaining conspecifics served as contact animals (group A - N = 4; groups B-D - N = 3) (Tables 1, 2). The challenge virus was administered by intramuscular (IM) injection bilaterally in the masseter muscle at a volume of 0.5 ml per site. Upon infection, the animals were observed at least twice daily for a period of 261 days and the development of clinical signs was recorded (Fig. 3). According to the animal welfare protocol, immediately at the onset of the first clinical signs, the animals were to be sedated and humanely euthanized by a supervising veterinarian with an overdose of thiafentanil oxalate (10 mg/ml Thianil, Wildlife Pharmaceuticals, Windsor, USA). Clinical signs included bellowing, throwing head back, persistent swallowing movements, persistent licking, paresis and paralysis, hypersalivation or frothing at the mouth. Point survivors were ear-tagged, vaccinated parenterally against rabies and rehomed on the spot at the end of the observation period.

**Table 1:** Group composition and survival after infection/challenge.

study	group	number of animals					time point (day p.v.)	inoculation dose in TCID50/mL	survival	percentage
		total	removed	total	female	male				
transmission	High dose	4	—	4	4	—	0	10^5.3^	0/4	0%
	Low dose	4	—	4	4	—	0	10^3.3^	1/4	25%
	contacts	13	1	12	12	—	0	—	11/12	91.6%
vaccination	parenteral	12	3	9	9	—	56	—	9/9	100%
	DOA	13	3	10	7	3	56	10^5.3^	3/10	30%

**Table 2:** Individual immune response of infected and contact animals from the transmission study as measured by ELISA (% inhibition), RAPINA and RFFIT (IU/ml).

Animal	Status	Group	B0 (day of capture)				sero status	B1 (day 261p.i.)					Outcome		FAT/rabies
			ELISA	RAPINA	RFFIT	score		ELISA	RAPINA	RFFIT	score	sero status	survival	death (days p.i.)	
K06	removed		**−−**	**−**	**−−**	−5	NEG						NO	−26	n.t.
K03	control; low dose	**A**	**−−**	**−**	**−−**	−5	NEG						NO	247	**POS**
K02	control; high dose		**−−**	**−**	**−−**	−5	NEG						NO	13	**POS**
K01	contact		**−−**	**−**	**−−**	−5	NEG	−−	**−**	++−−	−3	NEG	YES		n.t.
K04	contact		**++−−**	**+**	**−−**	−1	NEG	−−	+	++	1	**POS**	YES		n.t.
K05	contact		**−−**	**+**	**++−−**	−1	NEG						NO	156	**POS**
K16	control; low dose	**B**	**++**	**+**	**++−−**	3	**POS**						NO	233	**POS**
K13	control; high dose		**−−**	**−**	**++−−**	−3	NEG						NO	16	**POS**
K07	contact		**−−**	**−**	**++−−**	−3	NEG	−−	**−**	++	−1	NEG	YES		n.t.
K08	contact		**−−**	**−**	**++−−**	−3	NEG	−−	+	++	1	**POS**	YES		n.t.
K09	contact		**−−**	**−**	**−−**	−5	NEG	−−	**−**	−−	−5	NEG	YES		n.t.
K15	control; low dose	**C**	**++**	**−**	**++−−**	1	**POS**						NO	93	**POS**
K12	control; high dose		**++−−**	**−**	**++−−**	−1	NEG						NO	12	**POS**
K10	contact		**−−**	**−**	**−−**	−5	NEG	−−	+	++	1	**POS**	YES		n.t.
K11	contact		**−−**	**−**	**−−**	−5	NEG						NO	99	NEG
K14	contact		**−−**	**−**	**−−**	−5	NEG	−−	**−**	++−−	−3	NEG	YES		n.t.
K21	control; low dose	**D**	**++−−**	**−**	**−−**	−3	NEG	++	+	++	5	**POS**	YES		n.t.
K18	control; high dose		**−−**	**+**	**++−−**	−1	NEG						NO	15	**POS**
K17	contact		**++**	**−**	**++−−**	1	**POS**	++	+	++	5	**POS**	YES		n.t.
K19	contact		**++**	**+**	**−−**	1	**POS**	++	+	++−−	3	**POS**	YES		n.t.
K20	contact		**++**	**+**	**−−**	1	**POS**	++	+	++	5	**POS**	YES		n.t.

Indeterminate values were considered within a range of variation around the presumed cut-off (0.5 IU/mL +/− 0.25 IU/mL for RFFIT and 40% + 4.41%/− 5.28% for BioPro ELISA) as described previously (Moore *et al*., 2017). Final rating of the serological status of an individual serum followed a scoring scheme. Every + received a score of +1 and every − a score of −1. The total score was calculated by adding the values; a negative score (<0) was considered sero-negative and a positive score (>0) as sero-positive. Data are stratified according to infection status and the serological status at the day of capture (B0). Results of FAT testing are indicated (n.t. – not tested). All animals that survived the observation period of 261 days p.i. were vaccinated against rabies and rehomed.

### Vaccination studies

Twenty five Kudus were used for vaccination studies and held in 5 groups of four animals each and one group of five animals (Table 1). Prior to vaccination animals were allowed to adapt to captivity for 24 days. Ten animals (7 females and 3 males) in three groups received approx. 5.0 ml of the oral rabies virus vaccine construct SPBN GASGAS (10^8.1^ FFU/ml) by direct oral administration (DOA). As positive controls, 9 female Kudus divided over 3 groups received 2.0 ml of a commercial inactivated rabies vaccine (Rabisin, Merial, France) by the IM route (Table 1). All vaccinated animals were challenged on the same day together with the animals from the transmission study (56 days post vaccination (p.v.)) with the high dose (10^5.3^ TCID_50_) Kudu RABV isolate using the same route of administration and following the same termination criteria as described above. Survival of vaccinated animals was followed over a study period of 183 days post infection (p.i.) (Fig. 3).

### Sampling

Serum samples (B) were collected at different time points prior, p.v. and p.i. to investigate the development and kinetics of rabies induced antibodies (Fig. 3). Initial blood sampling was on the day of capture (B0); additional blood samples were taken on day 28 p.v. (B1) and 56 (B2) p.v. (vaccination study) as well as on day 261 p.i. (B1) and day 183 p.i. (B3) from contact and surviving animals of the challenge infection in the transmission and vaccination study, respectively (Fig. 3).

For any manipulation (e.g. sampling, vaccination), animals were always immobilized and sedated with a combination of 6–8 mg of thiafentanil oxalate (10 mg/ml Thianil, Wildlife Pharmaceuticals, Rocky Drift, White River, South Africa) and 100 mg azaparone (Kyron) reversed with 80 mg naltrexone hydrochloride (50 mg/ml Trexonil, Wildlife Pharmaceuticals). Sedation was carried out using a dart gun with X-Caliber CO_2_ operated dart projector with syringe darts of 2 ml with 14 GA × 25 mm needle (Pneu-Dart Inc., Williamsport, PA, USA).

### Viruses

For DOA an oral vaccine virus strain with a very high safety profile was used. The vaccine strain SPBN GASGAS licensed for foxes and raccoon dogs^19^ is derived from SAD L16, a cDNA clone of the oral RABV vaccine strain SAD B19. SPBN GASGAS lacks the pseudogene (ψ) and contains an additional identical RABV glycoprotein (G) which both show alterations at amino acid positions 194 and 333; position 194-AAT [Asn] → TCC [Ser], position 333-AGA [Arg] → GAG [Glu]^20^. It is postulated that the overexpression of the RABV G increased not only its efficacy but also its safety profile by reducing potential risk of reversion to virulence and enhancement of apoptosis^21,22^. The vaccine virus was propagated on BHK21 BSR Cl13 cells and harvested after 48 hours. The cell-cultured vaccine material was subsequently 5 times ultrafiltrated and stabilized (140 ml antigen + 60 ml GS8) to reach a final titre of 10^8.1^ FFU/ml.

The challenge RABV virus used (Lab ID 23079) was initially isolated from the brain of a naturally infected Kudu (240K09, GenBank accession JX473841) and genetically characterized^15^. After three serial passages on mouse neuroblastoma cells (NA42/13), the inoculum had a titre of 10^3.3^ MICLD_50_/ml and 10^5.3^ TCID_50_/ml when assayed by intracerebral inoculation of mice and cell culture, respectively. Verification of the genetic identity of the RABV isolate after passaging in cell culture using next generation sequencing^23^ revealed an additional insertion of three nucleotides at positions 2475–2477 (AAC) in the intergenic region between the phosphoprotein (P) and matrix protein (M) gene. This specific sequence variation was confirmed by conventional Sanger sequencing of two independent PCR amplicons (Supplementary material) and let to the extension of the 6 A transcription stop signal by 2 additional As, while the following intergenic region starts with an additional C comprising 7 nucleotides (nt).

### Ethical approval

This study was conducted under general Permits 101631, 101835, 101825, 101826 for (i) capturing, transport and keeping of game for commercial purposes and (ii) research in regard to problem animals under the Nature Conservation Ordinance 4 of 1975 as well as a research permit (Permit No 1984/2014 and 2152/2016) issued by the Namibian Ministry of Environment and Tourism (MET). The experimental holding facility conformed to the requirements of the Namibian MET for captive game. Both the experimental holding facility as well as the standard operating procedures for maintaining quarantine conditions was approved by the Directorate of Veterinary Services of the Ministry of Agriculture, Water and Forestry (MAWF). The methods were carried out in accordance with the relevant guidelines and regulations.

### Informed consent

The experiments did not involve human participants (including the use of human tissue samples). Therefore, informed consent is not needed.

### Diagnostic Assays

The presence of RABV antigen in brain tissue of Kudus was detected by fluorescent antibody test (FAT) as described previously^24^ using both polyclonal (OIE-RL Onderstepoort, South Africa) and commercial FITC- labeled monoclonal (SIFIN, Berlin, Germany) anti-rabies antibodies. Defined positive (PC, positive fox brain) and negative controls (NC, naïve cattle brain) were included in every test run. FAT indeterminate results were confirmed by realtime polymerase chain reaction (rt-qPCR)^25^ and the rabies tissue culture infection test (RTCIT)^26^, the latter with three consecutive passages to confirm a negative result.

Sera were tested for the presence of rabies specific antibodies using three different serological assays with modified cut-offs. Virus neutralizing antibodies (VNAs) were detected in a modified rapid fluorescent focus inhibition test (RFFIT) essentially as described^27^ using the calibrated WHO international standard immunoglobulin (2nd human rabies immunoglobulin preparation, National Institute for Standards and Control, Potters Bar, UK) adjusted to 0.5 international units (IU) and a naive bovine serum as PC and NC, respectively. VNA titres were calculated using inverse interpolation as described and expressed in international units (IU/mL)^28^. Presence of rabies specific binding antibodies were tested using a commercial blocking ELISA (BioPro Rabies ELISA, Czech Republic) strictly following manufacturer’s instructions. A study evaluating this ELISA kit found 100% specificity and 95.5% sensitivity with fox and raccoon dog sera; and 95% concordance with an assay measuring VNAs (the Fluorescent Antibody Neutralization Test)^29^. None of the serological assays employed in this study have been fully evaluated with Kudu sera, and a previous publication has demonstrated variable cut-off levels per species^27^. To account for lack of a unique identifiable cut-off in the assays for Kudu sera, besides sero-positive and sero-negative results indeterminate values were considered within a range of variation around the presumed cut-off of positivity (0.5 IU/mL +/− 0.25 IU/mL for RFFIT and 40% + 4.41%/−5.28% for BioPro ELISA) as described previously^27^. As a versatile and practical method for measuring rabies specific antibodies p.v., additionally the RAPINA test^30^ was applied as a 3^rd^ method by following instructions of the manufacturer. Final scoring if a serum sample was deemed antibody negative or positive was based on a weighted analysis of all results obtained by the 3 different assays without giving an absolute value to it. ELISA and RFFIT results were given more weight (++; −−; ++/−−) than the RAPINA test (+, −, +/−), for the latter is not considered a standard test for rabies serology yet; whereby ++ or + is sero-positive, −− or − is sero-negative and +/− or ++/−− indeterminate. Every + received a score of +1 and every − a score of −1. The total score was calculated by adding the values; a negative score (<0) was considered sero-negative and a positive score (>0) as sero-positive.

FAT testing was done in parallel at the Central Veterinary Laboratory (CVL) Windhoek and the Friedrich-Loeffler-Institut (FLI), Greifswald-Insel Riems, while rt-qPCR, RTCIT and all serological assays were exclusively conducted at FLI after the end of the experimental study.

### Statistical Analyses

To infer statistical differences in the serological results, the Fisher’s exact test was applied, whereas for survival rates the Mantel-Cox test (log-rank test) was used as implemented in GraphPad Prism version 7.00 (GraphPad Software, La Jolla California USA), with p-values < 0.05 considered significant.

## Results

Because of illnesses and severe injuries (fractures) as a result of capture stress, 7 animals had to be removed at various times throughout the study; one animal (K06) in the transmission group and 6 animals (K26, K25, K30, K37, K44, K45) in the vaccination groups between 26 and 78 days prior to infection (Tables 1–3). As a release of those animals back into the wild could not be justified, for reasons of animal welfare they had to be humanely euthanized. None of the animals showed clinical signs suggestive of rabies.

**Table 3:** Individual immune response of animals from the vaccination study immunized DOA and IM as measured by ELISA (% inhibition), RAPINA and RFFIT (IU/ml).

animal	Status	B0 (day of capture)					B1 (day 28 p.v.)					B2 (day 56 p.v.)					B3 (day 183 p.i.)					Outcome		
		ELISA	RAPINA	RFFIT	score	serostatus	ELISA	RAPINA	RFFIT	score	serostatus	ELISA	RAPINA	RFFIT	score	serostatus	ELISA	RAPINA	RFFIT	score	sero status	survival	death (days p.i.)	FAT/rabies
K22	IM	−−	+	++−−	−1	NEG	++	+	++	5	POS	++	+	++	5	POS	++	+	++	5	POS	YES		n.t.
K23	IM	−−	−	−−	−5	NEG	++	+	++	5	POS	++	+	++	5	POS	++	+	++	5	POS	YES		n.t.
K24	IM	−−	−	−−	−5	NEG	++	+	++	5	POS	++	+	++	5	POS	++	+	++	5	POS	YES		n.t.
K26	removed	−−	−	−−	−5	NEG																NO	−78	n.t.
K27	IM	++	−	−−	−1	NEG	++	+	++	5	POS	++	+	++	5	POS	++	+	++	5	POS	YES		n.t.
K29	IM	++−−	−	++−−	−1	NEG	++	+	++	5	POS	++	+	++	5	POS	++	+	++	5	POS	YES		n.t.
K30	removed	++−−	−	−−	−3	NEG																NO	−78	n.t.
K31	IM	−−	+	−−	−3	NEG	++	+	++	5	POS	++	+	++	5	POS	++	+	++	5	POS	YES		n.t.
K32	IM	++	+	++−−	3	POS	++	+	++	5	POS	++	+	++	5	POS	++	+	++	5	POS	YES		n.t.
K25	removed	++	−	++−−	1	POS																NO	−78	n.t.
K28	IM	++−−	+	++	3	POS	++	+	++	5	POS	++	+	++	5	POS	++	+	++	5	POS	YES		n.t.
K33	IM	−−	+	++	1	POS	++	+	++	5	POS	++	+	++	5	POS	++	+	++	5	POS	YES		n.t.
K34	DOA	−−	−	−−	−5	NEG	++−−	−	−−	−3	NEG	−	−	++−−	−3	NEG						NO	12	POS
K35	DOA	−−	+	−−	−3	NEG	++	+	++−−	3	POS	++	+	++	5	POS						NO	26	POS
K36	DOA	++−−	+	−−	−1	NEG	++	+	++	5	POS	++	+	++−−	3	POS						NO	15	POS
K37	removed	−−	−	−−	−5	NEG	++−−	−	++−−	−1	NEG											NO	−35	n.t
K38	DOA	−−	−	++−−	−3	NEG	−−	−	++−−	−3	NEG	−−	−	++−−	−3	NEG						NO	16	POS
K39	DOA	−−	−	−−	−5	NEG	−−	−	−	−5	NEG	−−	−	++−−	−3	NEG						NO	13	POS
K40	DOA	−−		−−	−4	NEG	−−	−	++−−	−3	NEG	−−	−	−−	−5	NEG	++	+	++	5	POS	YES		n.t.
K41	DOA	−−	+	−−	−3	NEG	−−	−	++−−	−3	NEG	−−	−	++−−	−3	NEG						NO	14	POS
K42	DOA	−−	−	++−−	−3	NEG	−−	−	++−−	−3	NEG	−−	−	++−−	−3	NEG						NO	18	POS
K43	DOA	−−	−	−−	−5	NEG	−−	−	++−−	−3	NEG	−−	−	++−−	−3	NEG	++	−	++	5	POS	YES		n.t.
K44	removed	−−	−	−−	−5	NEG																NO	−66	n.t.
K45	removed	−−	−	−−	−5	NEG																NO	−78	n.t.
K46	DOA	++−−	+	++−−	1	POS	++	−	−−	−1	NEG	++−−	−	−−	−3	NEG	++	+	++	5	POS	YES		n.t.

Indeterminate values were considered within a range of variation around the presumed cut-off (0.5 IU/mL +/− 0.25 IU/mL for RFFIT and 40% + 4.41%/− 5.28% for BioPro ELISA) as described previously (Moore et al., 2017). Final rating of the serological status of an individual serum followed a scoring scheme. Every + received a score of +1 and every – a score of −1. The total score was calculated by adding the values; a negative score (<0) was considered sero-negative and a positive score (>0) as sero-positive. Data are stratified according to the serological status at the day of capture (B0). Results of FAT testing are indicated (n.t. – not tested). All animals that survived the observation period of 183 days p.i. were revaccinated and rehomed.

### Transmission studies

**Figure 4:**
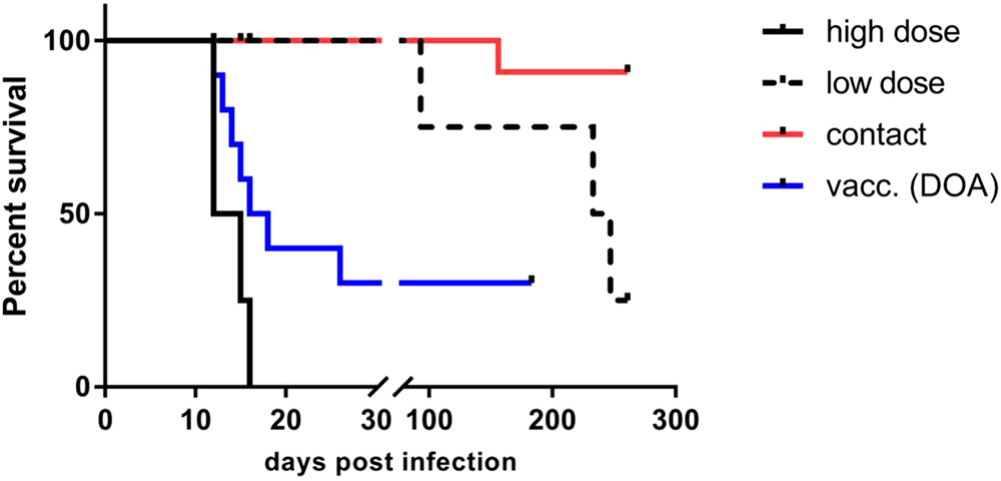
Survival curves of Kudu infected with a high dose (black solid line) and low dose (black dashed line), contact animals from the transmission group (red line) and the Kudu vaccinated by direct oral application (DOA, blue line). The median incubation period for high and low dose infected Kudus was 13.5 days and 233 days, respectively. Among animals from the DOA group that succumbed to rabies the median incubation period was 15 days. The difference in the survival of vaccinated vs. high dose infected animals from the transmission study was below the statistical level of significance (Log-rank/Mantel-Cox test, p = 0.0786).

While all four animals (K02, K12, K13, K18) inoculated with the high dose Kudu RABV isolate (10^5.3^ TCID_50_) were seronegative at B0 and succumbed to infection between day 12 and 16 p.i., all but one low dose infected (10^3.3^ TCID50) Kudus (K03, K15, K16) died or had to be euthanized between day 93 and 245 p.i. (Table 1, Fig. 4). Two of the latter animals (K15, K16) had rabies specific antibodies at B0 (Tables 2, S2). The Kudu (K21) that survived the low dose challenge virus infection seroconverted at day 261 p.i. Clinical signs included swallowing movements associated with moderate salivation, paresis, and mild frothing at the mouth in two cases. The remaining animals in the group mostly tried to stay away from and avoid contact with those displaying clinical signs. Six animals that succumbed to the infection were FAT positive while two were regarded FAT inconclusive, with the latter two being positive in RT-qPCR. All but two of the contact animals survived the 261 days observation period. Two of 12 contact animals died 99 (K11) and 156 days p.i. (K05), however, only in the latter were viral antigen and RNA detected by FAT and PCR, respectively (Tables 1, 2, Fig. 4). Sequencing of the RABV isolate of the FAT positive contact animal revealed a 100% sequence identity with the challenge virus including the insertion.

Of the 11 survivors (Tables 1, 2, Fig. 4), three contact animals (K17, K19, K20) were rated antibody positive at B0 and remained seropositive during the observation period of 261 days. Contrastingly, three out of 8 surviving contact animals (K04, K08, K10) were considered seronegative at the beginning of the study but seroconverted (Tables 2, S2).

### Vaccination studies

**Figure 5:**
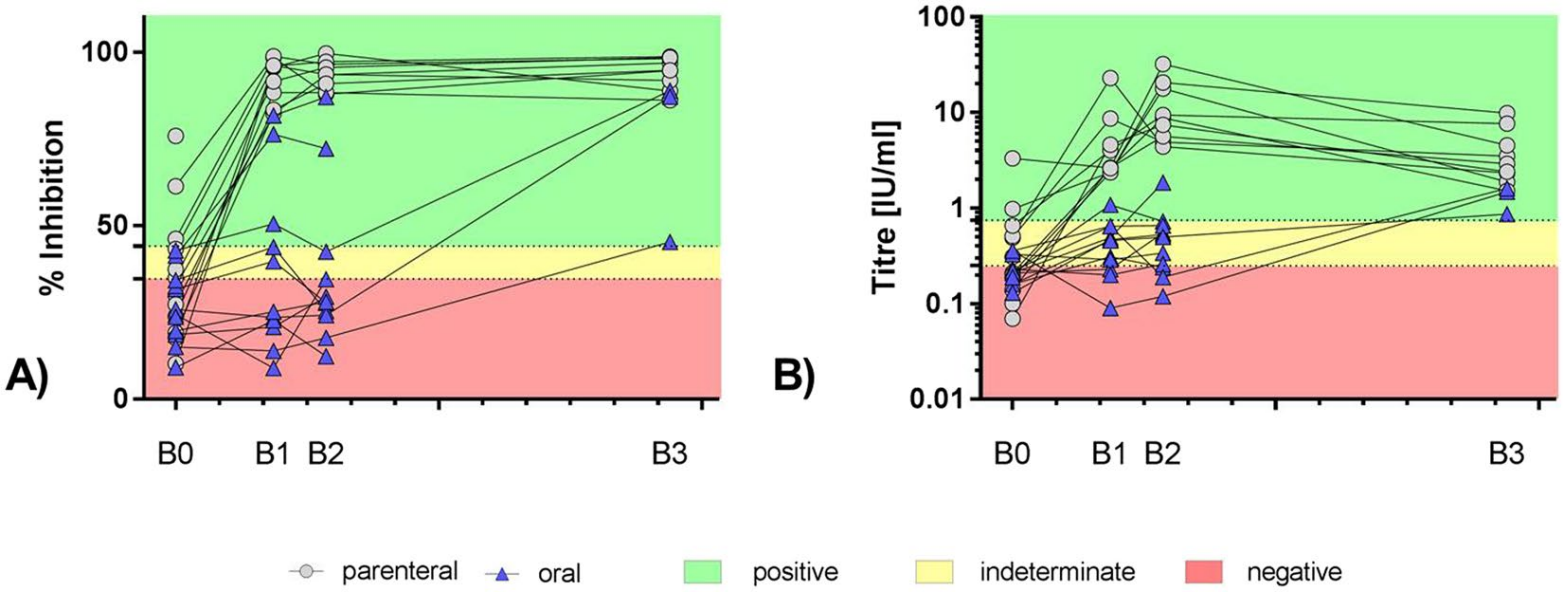
Graphical display of individual serological data from parenterally (full grey circles) and orally (blue triangles) vaccinated Kudus using ELISA (**A**) and RFFIT (**B**) over the course of the experimental study. B0 = blood sample at day of capture; B1 = day 28 p.v., B2 = day 56 p.v.; B3 = day 183 post infection (challenge). The interpretation of the values as positive, indeterminate and negative are indicated.

In the parenteral group, all 9 Kudus vaccinated IM developed a strong immune response as measured by the three independent assays and survived challenge infection (Table 1, Fig. 4), though 3 animals (K28, K32, K34) were already seropositive at the time point of capture (Tables 3, S3). Almost complete blocking in the ELISA was observed in sera from IM vaccinated Kudus at all time points p.v. with ELISA mean percent blocking (PB) values > 92% at days 28, 56, resembling a 2.9–3.0 increase compared to day 0. Contrastingly, with 4.18 and 9.90 IU/ml at day 28 and 56 p.v., respectively, the geometric mean titres (GMT) of VNAs of the IM vaccinated animals as measured by RFFIT increased 13.1 (day 28) – 31.0 fold (day 56) fold compared to day 0. While 183 p.i. the ELISA mean PB values almost remained the same, the GMT of VNAs decreased to levels as obtained at day 28 p.v. but were still 11 fold higher than at day 0 (Tables 3, S3, Fig. 5).

There was a significant difference (Fisher’s exact test, p = 0.0031) in the parenteral versus the DOA vaccinated group. In general, with < 31.4% the DOA vaccinated animals showed only a 1.3 fold increase in ELISA mean PB values at days 28 and 56 p.v. compared to day 0 and remained below the ELISA cut-off of positivity. Though there was a 1.7–2.0 fold increase compared to day 0 the GMT of VNAs of orally vaccinated animals remained below 0.5 IU/ml until challenge (Tables 3, S3, Fig. 5).

Only 3 of 10 Kudus (K40, K43, K46) vaccinated by the oral route survived the challenge (30%) (Table 2, Fig. 4). While two of these animals remained seronegative prior to infection, one Kudu (K46) had detectable levels of antibodies at the time point of capture but turned seronegative at days 28 and 56 p.v.. After challenge infection all three survivors developed high levels of rabies specific antibodies; the GMT of these individual animals increased to 1.27 IU/ml at day 183 p.i., resembling a 5.8 and 2.9 fold increase compared to day 0 and day 56 p.v., respectively (Tables 3, S3, Fig. 5). Of the 7 initially seronegative (B0, time point of capture) DOA vaccinated Kudu that succumbed to infection, two animals (K35, K36) developed rabies specific antibodies p.v.. All animals died between 12 and 26 days p.i. (Table 1, Fig. 4). Six animals were FAT-positive, while one FAT-negative animal tested positive in rt-PCR. There was no (Log-rank/Mantel-Cox test, p = 0.0786) difference in the survival curves of orally vaccinated vs. high dose infected animals from the transmission study.

## Discussion

There is no other country in the world with such a large population of Kudus than Namibia. The high population density as a result of game farming is thought to favor rabies epidemics among this species. Experiments involving large, highly stress-sensitive wild-caught species like Kudus are typically limited by space and cost. Also, the lack of suitable research facilities able to house these large antelopes under quarantine conditions restricted this study to a relatively remote holding site that is normally used for temporary housing of wild game for sale and auctioning, hence complicating observations, sampling, sampling storage and – transportation. The precautions and safety measures specifically developed for the purpose of the study were of high standard and have proven to be useful in preventing contact to free-roaming wildlife in adjacent areas (Fig. 2).

While the relatively short incubation periods of 12–26 days observed after high dose infection are within the lower bounds of the expected range and are comparable to those of carnivorous reservoir species, Kudus and cattle^12,19,31–34^, the extremely long incubation periods of 93–245 days after low dose infection are remarkable, particularly against the background of the same route of infection and the fact that the difference in viral dose was just two log 10 steps (Fig. 4). However, long incubation periods using a similar dose have also been reported for skunks, golden jackals, raccoons and foxes^35–37^. In addition, the outcome of the transmission studies showed that depending on the viral dose Kudus are able to survive an infection with RABV and hence, confirm previous observations^12^. The animal (K21) that survived infection never showed clinical signs, indicating that the virus was recognized and cleared by the immune system either prior to movement into peripheral nerves or prior to infection of the central nervous system. This finding may also explain why several animals were sero-positive at the day of capture (Tables 2, 3, S2, S3).

The sheer magnitude of the epizootic and phylogenetic data are believed to support horizontal transmission and maintenance of a rabies cycle within this species^1,8^. However, despite anecdotal evidence of horizontal rabies transmission among Kudus, to this day this hypothesis has still not been definitively confirmed in a larger experimental setting. The hypothesis is based on the observation of hypersalivation and high viral titres in saliva of rabid Kudus, their grooming habits and in particular experimental studies^11,12^. Although artificial exposure to infection showed that 2 out of 4 Kudus died of rabies after experimental infection by instillation of infected saliva onto their buccal mucosae, intranasal (i.n.) infection cannot be excluded to have provoked the disease because the saliva was also instilled into the nasal cavity at the same time^12^. In fact, i.n. administration is a very effective way of delivering RABV directly to the brain via the olfactory nerve^38,39^. Our study seems to corroborate the possibility of horizontal transmission as the virus isolated from the contact animal possessed the unique marker of the challenge strain used. Since the length of the stop signals and intergenic regions affect polymerase termination and downstream re-initiation^40^ potential effects of this three nt insertion in the intergenic region between P and M gene on virus replication cannot be excluded. However, since selection of the additional insertion in the course of limited cell culture passages is more likely to support virus replication and the glycoprotein has been identified as the major pathogenicity factor^41^, major effects on the virulence of the challenge virus are unlikely.

Also, the observation that 3 of 8 contact animals (K04, K08, K10) considered seronegative at the time point of capture seroconverted 261 days p.i. (Tables 2, S2) could be considered evidence for exposure with infectious saliva from inoculated Kudus which succumbed to the disease. Unfortunately, the relationship between dose and strength of priming using street RABVs is not well characterized in both bats and other wildlife^42^. Although our experimental settings allowed natural social and feeding behavior to the greatest possible extent, Kudus had to be humanely euthanized early after showing clinical signs. This may have limited the likelihood of successful onward transmission leading to horizontally acquired rabies. The fact that only one of 12 contact animals (K05) died of rabies needs careful interpretation. If our observations resemble naturally occurring infections it would suggests that the extent of horizontal transmission under field conditions is less than is actually believed. Even if the transmission rate was underestimated and acquired seropositivity of contact animals (K04, K08, K10) (Tables 2, S2) was also considered as evidence of transmission, the latter being highly debatable, local horizontal transmission cannot plausibly explain the recent epidemics and rapid spread of the disease in Kudus over large territories. In this sense, long incubation periods might add to the situation explaining why rabies in Kudu is resurgent after months in areas where it is believed to have disappeared. Interestingly, high number of cattle rabies cases and other small ruminants in areas where Kudu rabies is endemic (unpublished) also bring into question the role of the Kudu as a sole reservoir. While the phylogenetic work of Scott and co-workers^15^ seem to support evidence for independent horizontal transmission among Kudus, this is contradicted by an earlier study which suggest that jackal and Kudu may form part of the same epidemiological cycle of rabies in Namibian wildlife^43^. From an epidemiological point of view, a combination of spill-over events and perhaps locally restricted horizontal transmission cannot be excluded, however, has not been considered or discussed thus far. Also, other epidemiological aspects including management, translocation, natural movement behavior and other parameters would definitely need a more thorough investigation and consideration to unravel the mystery of Kudu rabies in Namibia.

Expanding the concept of oral vaccination against rabies as successfully developed for wild carnivores to Kudus seems to be a feasible approach considering that commercial products for oral immunization of ruminants already exist; e.g. for immunization of calves in controlling diarrhea caused by bovine rotavirus and – coronavirus (Calf-Guard®, Zoetis). However, mucosal immunization through oral delivery is often compromised by antigen degradation in the stomach, especially considering the complex gastro-intestinal tracts of larger ruminants^44^. Therefore, to assure antigen uptake at Peyer’s patches of the intestine additional substances are often added to oral vaccine formulations protecting the antigen against enzymatic and proteolytic degradation. However, the gut is not the only site with mucous-associated lymphoid tissue (MALT) containing lymphoid follicles and M-cells. MALT can also be found in nasal and oral cavities, whereby tonsils form a major component in the latter. Recent studies have indicated that for oral vaccination of meso-carnivores against rabies the palatine tonsils are a major site of vaccine uptake^45–47^. Palatine tonsils are also present in ruminants like cattle, sheep and goats^48^. Experimental studies in wild ruminants like white-tailed deer (*Odocoileus virginianus*) showed that DOA of BCG-vaccine targeting the pharyngeal lymphoid tissue was able to induce an immune response against bovine tuberculosis^49^. Also, oral administration of heat-inactivated *Mycobacterium bovis* to red deer (*Cervus elaphus*) induced some level of protection against bovine tuberculosis^50^. In our proof-of-principle experiment, 3 of 10 animals orally vaccinated with SPBN GSAGAS survived a severe rabies challenge infection with a high challenge dose (Table 1, Fig. 4). Even though no statistical significance in survival between unvaccinated and DOA vaccinated animals was found, the survival of Kudu suggest that oral vaccination may elicit a protective immune response in this species.

Interestingly, none of these 3 animals (K40, K43, K46) that survived had detectable levels of antibodies in any of the assays (ELISA, RAPINA, RFFIT) p.v., however, the strong immune response p.i. is clear evidence for a prime-boost effect (Tables 3, S3, Fig. 5). It must be mentioned that the vaccine virus was evenly distributed in the oral cavity of the Kudus using a needleless syringe simulating release of vaccine when chewing on a vaccine sachet and the palatine tonsillar crypts as potential uptake site were not specifically targeted. However, unfortunate spillage of vaccine virus could not be prevented as most of the anesthetics including thiafentanil oxalate induce salivation in wild herbivores and in combination with grinding often produce frothing around the mouth^51^. So this per se makes DOA in these species difficult. On the other hand, one can argue that 30% survival in orally vaccinated Kudus (Table 1, Fig. 4) after challenge does not appear very convincing. However, it is the first time that such a proof-of-concept study has ever been conducted in an herbivorous species such as Kudus. The vaccine strain SPBN GASGAS has shown to be efficacious in several animal species like red foxes (*Vulpes vulpes*), raccoon dogs (*Nyctereutes procyonoides*), raccoon (*Procyon lotor*) and small Indian mongoose (*Herpestes auropunctatus*)^19,52,53^. However, striped skunks (*Mephitis mephitis*) seem to be rather refractory to vaccination through the oral route in a vaccine virus titer dependent manner^47^. It cannot be excluded that this could also apply to Kudus.

Performance of serological assays used and the interpretation of the serological results obtained presented problems. While the RFFIT is an OIE and WHO recommended test for detection of VNAs as for response to vaccination in humans and animals (particularly in connection with international travel of pets), the ELISA and RAPINA test have been developed for qualitative and semi-quantitative detection of rabies antibodies in foxes and raccoon dogs^29^, and humans and dogs^30^, respectively. None of these serological assays, however, have been validated for Kudus. Therefore, we modified the thresholds of positivity by introducing an additional ‘grey zone’ for inconclusive or indeterminate results based on analysis by Moore *et al.*^27^. The subsequent weighted scoring allowed a clear identification of sera as being seropositive and negative without giving an absolute value to it and by avoiding classifying sera as indeterminate. Of note, additional results from another ELISA (Platelia II, Bio-Rad, Tables S2, S3)^54,55^ was excluded from the analysis as Protein A does not bind well to IgG from bovine and equine species^56^.

Using this approach, only the results obtained p.v. with the animals vaccinated by the parenteral route showed a perfect correlation between seroconversion and protection for both serologic assays. However, for the animals that received the vaccine by the oral route, the serology results are more difficult to interpret (Tables 3, S2, Fig. 5) (see above). While 3 DOA vaccinated but seronegative animals survived challenge, two Kudus with supposedly acquired or preexisting antibodies at the day of challenge (K35, K36) succumbed to infection indicating a non-protective immunity (Tables 3, S3). Perhaps, the measured humoral antibody response prior to challenge is not of sufficient specificity or not associated with cellular immunity to prevent a lethal infection. The results of this study somewhat corroborate results obtained in studies on rabies repeated challenge in bats^42^ and suggest that a single exposure may not protect Kudus significantly against subsequent infections. These findings also underscore that diagnostic assays validated (specificity and sensitivity) for samples from certain species are not automatically suitable for samples from other species, as was recently shown for serology by Moore *et al*.^27^.

## Conclusions

Kudus can be vaccinated by the oral route and protected against a subsequent rabies infection, although it seems that they are rather refractory to this route of vaccine administration. In any case, further studies need to be initiated to optimize oral vaccine uptake and delivery of this 3^rd^ generation attenuated oral rabies vaccine. Alternatively, recombinant rabies virus vaccines expressing the RABV glycoprotein could also be considered in future studies^57^. For the time being the minimum effective titer of both attenuated and recombinant vaccine viruses required to efficiently immunize the animals is not known yet. Hence, further research should investigate how vaccine uptake effectiveness can be improved, for example by increasing vaccine titre, vaccination intervals or adding muco-adhesive substances. Attractive baits for oral vaccination of Kudus have been developed already^58^, however, bait delivery systems need to be optimized in case vaccine potency can be enhanced in this species. Also, validation of serological assays for Kudus is required to make better informed decisions on the immune status.

## Electronic supplementary material

Supplementary Dataset
