# Supplementary material for: Experimental screening studies on rabies virus transmission and oral rabies vaccination of the Greater Kudu (Tragelaphus strepsiceros)
Source: Sci Rep. 2018 Nov 9;8:16599. doi: 10.1038/s41598-018-34985-5 (PMC6226427; doi:10.1038/s41598-018-34985-5)
Supplement: Supplementary file 1 — Supplementary Dataset [file 41598_2018_34985_MOESM1_ESM.pdf]

Experimental screening studies on rabies virus transmission and oral rabies vaccination of the Greater Kudu (*Tragelaphus strepsiceros*)

Rainer Hassel[1], Ad Vos[2], Peter Clausen[3], Susan Moore[4], Jolandie van der Westhuizen[5], Siegfried Khaiseb[5], Juliet Kabajani[5], Florian Pfaff[6] , Dirk Höper[6], Boris Hundt[2], Mark Jago[1], Floris Bruwer[7], Pauline Lindeque[7], Stefan Finke[8], Conrad M. Freuling[8], Thomas Müller[8]*

Supplementary material:

PCR and Sanger sequencing

In order to confirm the genetic changes observed for the passaged inoculum virus by high-throughput sequencing (insertion of the three nucleotides AAC at positions 2475-2477 relative to JX473841) two independent PCRs were designed. Briefly, primer sequences (Table S1) were deduced using Primer3web (version 4.0.0) and PCR was carried out using SuperScript III one-step RT-PCR system with Platinum Taq high-fidelity polymerase (Invitrogen, Carlsbad, CA, USA), according to the manufactures instructions and using the same RNA as input as for high-throughput sequencing. Subsequently, PCR products were separated on an ethidium bromide stained agarose gel (1.5%) and visible bands were cut out. DNA was extracted from gel slices using the NucleoSpin gel and PCR clean-up kit (Macherey-Nagel, Düren, Germany). Invoking either the forward or reverse binding primer, a sequencing PCR was set up using BigDye terminator v1.1 cycle sequencing kit (Applied Biosystems, Darmstadt, Germany). The resulting PCR products were column cleaned using the NucleoSEQ kit (Macherey-Nagel, Düren, Germany) and sequenced using the 3130 genetic analyzer (Applied Biosystems).

Table S1: Primers used for PCR. Positions are relative to reference JX473841.

Primer name	Direction	Sequence	Position	Product size in bp
Kudu_FW1	fwd	AAGGTGTGTACTGGGATGGG	2,290 -> 2,309	344
Kudu_RV1	rev	GTGAGTTCTTTCAGCGGGAC	2,632 -> 2,613	
Kudu_FW2	fwd	AGATCGCTCACCAGATTGCT	2,112 -> 2,131	487
Kudu_RV2	rev	AAGCCACAGGTCATCGTCAT	2,597 -> 2,578	

Table S2.Individual values of immune response of infected and contact animals from the transmission study as measured by ELISA (% inhibition), RAPINA and RFFIT (IU/ml). Indeterminate values were considered within a range of variation around the presumed cut-off (0.5 IU/mL +/- 0.25 IU/mL for RFFIT and 40% + 4.41%/- 5.28% for BioPro ELISA) as described previously (Moore et al., 2017). Data are stratified according to infection status and the serological status at the day of capture (B0). Results of FAT testing are indicated (n.t. – not tested). All animals that survived the observation period of 261 days p.i. were vaccinated against rabies and rehomed.

			B0 (day of capture)				B1 (day 261p.i.)				Outcome		
**Animal**	**Status**	**Group**	**ELISA**	**RAPINA**	**RFFIT**	**sero status**	**ELISA**	**RAPINA**	**RFFIT**	**sero status**	**survival**	**death (days p.i.)**	**rabies**
K06	removed		15.38	-	0.21	NEG					NO	-26	n.t.
K03	control; low dose		3.41	-	0.23	NEG					NO	247	**POS**
K02	control; high dose		10.12	-	0.09	NEG					NO	13	**POS**
K01	contact		5.81	-	0.08	NEG	7.95	-	0.36	NEG	YES		n.t.
K04	contact		39.45	+	0.12	NEG	30.78	+	1.74	**POS**	YES		n.t.
K05	contact	A	28.19	+	0.69	NEG					NO	156	**POS**
K16	control; low dose		45.33	+	0.38	**POS**					NO	233	**POS**
K13	control; high dose		25.12	-	0.45	NEG					NO	16	**POS**
K07	contact		16.71	-	0.31	NEG	13.18	-	0.84	NEG	YES		n.t.
K08	contact		33.12	-	0.88	NEG	27.17	+	8.46	**POS**	YES		n.t.
K09	contact	B	4.26	-	0.06	NEG	10.85	-	0.21	NEG	YES		n.t.
K15	control; low dose		73.90	-	0.32	**POS**					NO	93	**POS**
K12	control; high dose		41.12	-	0.63	NEG					NO	12	**POS**
K10	contact		10.15	-	0.13	NEG	-1.30	+	3.00	**POS**	YES		n.t.
K11	contact		19.66	-	0.04	NEG					NO	99	NEG
K14	contact	C	19.39	-	0.23	NEG	9.30	-	0.63	NEG	YES		n.t.
K21	control; low dose		35.01	-	0.05	NEG	46.96	+	1.04	**POS**	YES		n.t.
K18	control; high dose		18.55	+	0.39	NEG					NO	15	**POS**
K17	contact		49.11	-	0.57	**POS**	50.11	+	3.21	**POS**	YES		n.t.
K19	contact		82.26	+	0.07	**POS**	74.65	+	0.30	**POS**	YES		n.t.
K20	contact	D	92.57	+	0.14	**POS**	92.23	+	1.01	**POS**	YES		n.t.

Table S3. Individual values of immune response of animals from the vaccination study immunized DOA and IM as measured by ELISA (% inhibition), RAPINA and RFFIT (IU/ml). Indeterminate values were considered within a range of variation around the presumed cut-off (0.5 IU/mL +/- 0.25 IU/mL for RFFIT and 40% + 4.41%/- 5.28% for BioPro ELISA) as described previously (Moore et al., 2017). Data are stratified according to the serological status at the day of capture (B0). Results of FAT testing are indicated (n.t. – not tested). All animals that survived the observation period of 183 days p.i. were revaccinated against rabies and rehomed.

		**B0 (day of capture)**				**B1 (day 28 p.v.)**				**B2 (day 56 p.v.)**				**B3 (day 183 p.i.)**				**outcome**		
**animal**	**Status**	**ELISA**	**RAPINA**	**RFFIT**	**sero status**	**ELISA**	**RAPINA**	**RFFIT**	**sero status**	**ELISA**	**RAPINA**	**RFFIT**	**sero status**	**ELISA**	**RAPINA**	**RFFIT**	**sero status**	**survival**	**death (days p.i.)**	**rabies**
K22	IM	24.0	+	0.51	NEG	88.3	+	22.96	**POS**	88.3	+	4.42	**POS**	86.2	+	2.34	**POS**	YES		n.t.
K23	IM	19.2	-	0.14	NEG	82.3	+	2.67	**POS**	93.5	+	5.66	**POS**	96.9	+	2.92	**POS**	YES		n.t.
K24	IM	10.4	-	0.19	NEG	95.5	+	2.38	**POS**	99.6	+	17.88	**POS**	88.7	+	1.89	**POS**	YES		n.t.
K26	removed	29.9	-	0.10	NEG													NO	-78	n.t.
K27	IM	61.4	-	0.21	NEG	98.8	+	2.63	**POS**	96.5	+	8.81	**POS**	98.1	+	1.52	**POS**	YES		n.t.
K29	IM	35.1	-	0.66	NEG	91.5	+	4.03	**POS**	95.6	+	9.47	**POS**	98.4	+	7.69	**POS**	YES		n.t.
K30	removed	37.5	-	0.20	NEG													NO	-78	n.t.
K31	IM	17.8	+	0.07	NEG	96.1	+	4.63	**POS**	93.6	+	7.45	**POS**	91.8	+	2.41	**POS**	YES		n.t.
K32	IM	46.2	+	0.49	**POS**	98.4	+	8.65	**POS**	87.9	+	4.92	**POS**	94.6	+	3.51	**POS**	YES		n.t.
K25	removed	75.9	-	0.31	**POS**													NO	-78	n.t.
K28	IM	43.3	+	0.98	**POS**	96.0	+	2.38	**POS**	97.2	+	20.73	**POS**	98.6	+	9.89	**POS**	YES		n.t.
K33	IM	27.4	+	3.32	**POS**	83.4	+	2.65	**POS**	90.9	+	32.21	**POS**	94.7	+	4.56	**POS**	YES		n.t.
K34	DOA	31.8	-	0.21	NEG	39.8	-	0.23	NEG	27.9	-	0.55	NEG					NO	12	**POS**
K35	DOA	32.6	+	0.21	NEG	81.7	+	0.49	**POS**	87.0	+	1.85	**POS**					NO	26	**POS**
K36	DOA	41.4	+	0.21	NEG	76.3	+	1.08	**POS**	72.2	+	0.73	**POS**					NO	15	**POS**
K37	removed	34.3	-	0.16	NEG	43.9	-	0.31	NEG									NO	-35	n.t
K38	DOA	24.5	-	0.33	NEG	9.0	-	0.29	NEG	34.6	-	0.34	NEG					NO	16	**POS**
K39	DOA	18.6	-	0.23	NEG	20.8	-	0.20	NEG	29.6	-	0.26	NEG					NO	13	**POS**
K40	DOA	25.9		0.22	NEG	23.6	-	0.65	NEG	24.2	-	0.19	NEG	87.2	+	1.62	**POS**	YES		n.t.
K41	DOA	9.2	+	0.13	NEG	22.8	-	0.47	NEG	12.4	-	0.53	NEG					NO	14	**POS**
K42	DOA	19.8	-	0.36	NEG	25.2	-	0.65	NEG	28.1	-	0.66	NEG					NO	18	**POS**
K43	DOA	15.1	-	0.16	NEG	14.0	-	0.46	NEG	17.7	-	0.5	NEG	45.3	-	0.87	**POS**	YES		n.t.
K44	removed	23.8	-	0.19	NEG													NO	-66	n.t.
K45	removed	15.7	-	0.20	NEG													NO	-78	n.t.
K46	DOA	42.9	+	0.36	**POS**	50.6	-	0.09	NEG	42.5	-	0.12	NEG	88.9	+	1.48	**POS**	YES		n.t.
